# Joint association of dietary live microbe intake and depression with cancer survivor in US adults: evidence from NHANES

**DOI:** 10.1186/s12885-025-13699-8

**Published:** 2025-03-17

**Authors:** Dekui Jin, Tian Lv, Chengying Zhang, Yi Hu

**Affiliations:** 1 Medical School of Chinese PLA, Chinese PLA General Hospital, Beijing, 100853 China; 2 Department of Neurology, Zhuji Affiliated Hospital of Wenzhou Medical University, Shaoxing, Zhejiang China; 3 Department of General Practice, The Third Medical Center of Chinese PLA General Hospital, Beijing, 100039 China; 4 Senior Department of Oncology, the Fifth Medical Center of PLA General Hospital, Beijing, 100853 China

**Keywords:** NHANES, Dietary live microbe intake, Depression, Cancer survivor, Mortality

## Abstract

**Background:**

The mortality of cancer survivors is influenced by various factors. This study aims to investigate the relationship between dietary live microbe intake and depression with the mortality of cancer survivors among U.S. adults.

**Methods:**

This cross-sectional study utilized data from the National Health and Nutrition Examination Survey (NHANES) spanning from 2001 to 2018. Based on the classification by Sanders et al., foods were categorized by their levels of live microbes as follows: low (< 10^4 CFU/g), medium (10^4–10^7 CFU/g), and high (> 10^7 CFU/g). Using this classification and dietary questionnaire data, participants were divided into three groups: (1) low dietary live microbe intake (only low-level foods), (2) medium dietary live microbe intake (medium but not high-level foods), and (3) high dietary live microbe intake (any high-level foods). Additionally, foods classified as medium and high were combined into a “Medium-High” category. Cancer survivors were identified by their affirmative response to the question: “Have you ever been told by a doctor or other health professional that you had cancer or malignancy of any kind?” The Patient Health Questionnaire-9 (PHQ-9) was administered to assess depressive symptoms, with a score of ≥ 10 indicating depression. The study examined the independent and joint associations of dietary live microbe intake and depression with mortality outcomes in cancer survivors, employing Cox regression analysis adjusted for weights to calculate relative risk. Mediation analysis was conducted to evaluate the effect of PHQ-9 on the relationship between dietary live microbe intake and all-cause mortality in cancer patients.

**Results:**

During a median follow-up of 6.2 years, we identified a total of 605 all-cause mortality among participants, including 204 from cancer and 401 from non-cancer-related causes. The analysis showed that medium-high dietary live microbe intake was consistently associated with a lower risk of all-cause mortality (HR, 0.741; 95% CI, 0.602–0.912; *P* = 0.005) and non-CVD mortality (HR, 0.687; 95% CI, 0.545–0.866; *P* = 0.001) when compared to low dietary live microbe intake in adjusted models. Conversely, depression was linked to a higher risk of all-cause mortality (HR, 1.789; 95% CI, 1.281–2.473; *P <* 0.001) and non-CVD mortality (HR, 1.901; 95% CI, 1.249–2.793; *P* = 0.001) compared to individuals without depression. Notably, joint analyses revealed that low dietary live microbe intake was associated with the highest risk of all-cause mortality among cancer survivors who also experienced depression (HR, 3.122; 95% CI, 1.734–5.619; *P* <  0.001). Additionally, mediation analysis indicated that the PHQ-9 score mediated 18.4% of the association between dietary live microbe intake and all-cause mortality in cancer survivors mediation proportion 18.4%; 95% CI, 7.5-29.2%.

**Conclusions:**

Our results indicated that low dietary live microbe intake and depression are associated with an increased risk of non-CVD and all-cause mortality among cancer survivors. Additionally, the PHQ-9 score demonstrated a mediating effect on the relationship between dietary live microbe intake and all-cause mortality in this population.

**Supplementary Information:**

The online version contains supplementary material available at 10.1186/s12885-025-13699-8.

## Introduction

Cancer is characterized by the uncontrolled and abnormal growth of cells, resulting from genetic mutations or environmental factors [1]. It is the second leading cause of death worldwide [2]. In 2022, the International Agency for Research on Cancer (IARC) reported nearly 20 million new cancer cases and approximately 9.7 million cancer-related deaths. Additionally, projections indicate that new cancer cases could rise to 35 million by 2050 [3],leading to an increase in mortality rates. Among the key environmental factors associated with an increased cancer risk is diet [4]. A healthy diet may reduce the risk of developing cancer and lower the mortality risk for cancer survivors [5–9]. Research indicates that the consumption of live microbes can be beneficial to human health [10]. This is due to their ability to enhance intestinal function and reduce disease susceptibility by interacting with the resident gut microbiota [11]. Studies have shown that probiotic and fermented foods containing live microbes are inversely associated with cancer risk [12–16]. Additionally, live microbes are present in a variety of foods, including unpeeled fruits, vegetables, and meats [17]. However, the relationship between the intake of dietary live microbe and cancer mortality has not been thoroughly investigated. Furthermore, cancer survivors face an increased risk of mental health issues over time. Studies show that cancer survivors experience higher rates of depression, with prevalence estimates around 10% [18]. Depression has been linked to poorer survival outcomes in cancer patients [19–22]. Research indicates that probiotic and fermented foods containing live microbes can alleviate symptoms of depression [23, 24]. Consequently, we hypothesize that the intake of dietary live microbe may lower cancer mortality by reducing depression. Moreover, the relationship between dietary live microbe, depression, and cancer mortality is intricate and not well understood. Prior studies have largely concentrated on the separate effects of dietary patterns or depression on cancer mortality, neglecting their combined influence, particularly in relation to dietary live microbe [25–27].To date, it remains unclear whether there is a combined effect of dietary live microbe intake and depression on mortality risk among cancer survivors.

This study aimed to investigate both the independent and combined associations of dietary live microbe intake and depression with all-cause, cardiovascular disease (CVD), and non-CVD mortality among cancer survivors in the USA. Our findings may motivate cancer survivors, healthcare providers, and policymakers to raise awareness and implement targeted support initiatives to enhance patient outcomes.

## Materials and methods

### Study design and participants

This study utilized data from the National Health and Nutrition Examination Survey (NHANES) in the United States. The analysis followed NHANES analytical guidelines, which employ a multi-stage stratified systematic sampling design. NHANES consists of a series of surveys conducted by the National Center for Health Statistics (NCHS) to evaluate the health and nutritional status of a representative sample of the noninstitutionalized U.S. population.The NHANES dataset is composed of five primary components: demographics, dietary information, physical examinations, laboratory results, and questionnaire responses. The survey employs detailed interviews and thorough physical evaluations to gather relevant data. Data collection is crucial for informing public health policies and programs, evaluating the effectiveness of health and nutrition initiatives, and tracking the prevalence of various diseases and conditions. All NHANES protocols were approved by the National Center for Health Statistics Research Ethics Review Board, and informed consent was obtained from all study participants before their involvement. Moreover, all studies adhered strictly to the relevant protocols and regulations.For comprehensive information about NHANES data collection, interested individuals can refer to the published materials available at https://www.cdc.gov/nchs/nhanes.htm.

**Fig. 1:**
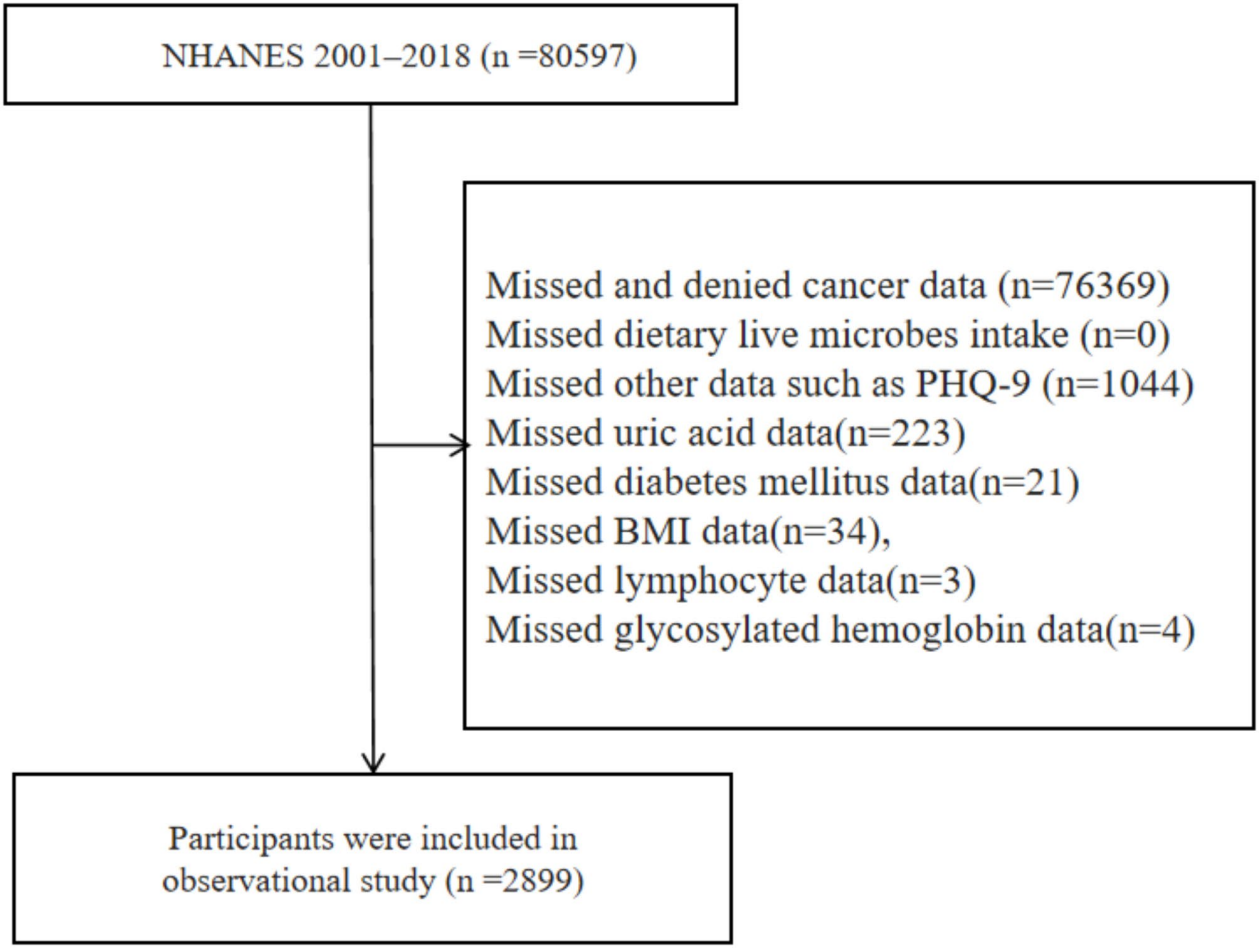
Flow chart of the study population

Between 2001 and 2018, a total of 80,597 individuals were initially registered in NHANES. The exclusion criteria included missed or denied cancer data (*n* = 76,369), and missing data on other variables such as PHQ-9 (*n* = 1,044), uric acid (*n* = 223), diabetes mellitus (*n* = 21), BMI (*n* = 34), lymphocyte counts (*n* = 3), and glycosylated hemoglobin (*n* = 4). Ultimately, 2,899 cancer survivor participants were included in this study(Fig. 1).

### Definition and estimation of dietary live microbe intake

Estimates of energy and nutrient intake were derived from 24-hour dietary recall data linked to the Food and Nutrient Database for Dietary Studies specific to the NHANES cycle, provided by the U.S. Department of Agriculture (USDA). The categorization of live microbes in foods was derived from research by Sanders et al.l [17]. To calculate the count of living microbes per gram in foods linked to the 9,388 food codes in the NHANES database, the assessment of each food by four experts(Maria L. Marco, Mary E. Sanders, Robert Hutkins, and Colin Hill) involved previous literature, authoritative reviews, and the known effects of food processing, including pasteurization, on the viability of microbes. Foods were divided into three categories according to the anticipated count of live microbes: low (< 10^4 CFU/g), medium (10^4–10^7 CFU/g), or high (> 10^7 CFU/g). These groups relate to pasteurized foods (< 10^4 CFU/g), unpeeled fresh fruits and vegetables (10^4–10^7 CFU/g), and unpasteurized fermented foods and probiotic supplements (> 10^7 CFU/g). The classification for each food was established through both intra- and inter-team consultations, with external input from Fred Breidt, a microbiologist at the USDA Agricultural Research Service. Prior research outlines the classification of each food in detail [17].

Since the method identifies live microbes in foods, it’s important to classify the levels of live microbes across the whole diet. Using the methodology from Sanders et al., participants were classified into three groups: (1) low dietary live microbe intake (consuming only low-level foods), (2) medium dietary live microbe intake (consuming medium but not high-level foods), and (3) high dietary live microbe intake (consuming any high-level foods). Additionally, we combined the amounts of medium-level and high-level foods consumed per person (in 100 g) and labeled this as “Medium-high”.

### Assessment of depression, cancer and covariates

Cancer survivors’ depressive symptoms over the two weeks prior to the survey were assessed using the Patient Health Questionnaire-9 (PHQ-9), which has been validated and found reliable in cancer patients [28, 29]. Nine items related to symptoms of depression are included in the PHQ-9: lack of interest, depressed mood, sleep disturbances, fatigue, appetite changes, feelings of worthlessness, lack of concentration, psychomotor agitation or retardation, and suicidal thoughts. Each item is rated on a scale from 0 (not at all) to 3 (almost daily), yielding a total score between 0 and 27, with higher scores indicating more severe depressive symptoms. Depressive symptoms were categorized as none (scores of 0–4), mild (scores of 5–9), or moderate to severe (scores of 10 or higher) [30]. An earlier study set a threshold score of 10 to detect patients exhibiting depressive symptoms: individuals scoring 9 or less were considered to have no clinically relevant depressive symptoms, whereas those scoring 10 or more were classified as having clinically relevant depressive symptoms [31]. This cutoff has been shown to have 88% sensitivity and 88% specificity for diagnosing major depression [32].

Details about the cancer histories of participants were obtained from: the “medical conditions” section of the NHANES database. Individuals who survived cancer were recognized by their positive response (“yes”) to the question: “Have you ever been told by a doctor or other health professional that you had cancer or malignancy of any kind?“.

Potential covariates were selected based on existing knowledge of the relationships between dietary live microbe intake, depression, and cancer mortality. In this study, specific covariates were analyzed for both descriptive and inferential statistics, including demographic factors, blood test results, and comorbid conditions. The demographic factors considered included age, gender (Male/Female), race (Black, White, Other), and Body Mass Index (BMI). The blood test results encompassed levels of blood urea nitrogen, albumin, neutrophil granulocyte count, hemoglobin, uric acid, total bilirubin, and HbA1c. Comorbid conditions included diabetes mellitus (DM), hypertension, and cardiovascular disease (CVD), based on self-reported physician diagnoses gathered during individual interviews using a standardized medical condition questionnaire.

### Mortality ascertainment

The outcomes examined in our study included all-cause mortality, cardiovascular disease (CVD) mortality, and non-CVD mortality within the study population. Death data for the follow-up population were sourced from the NHANES public-use linked death file, which was matched with the National Center for Health Statistics (NCHS) using the National Death Index (NDI) through a probability matching algorithm. The underlying causes of death were identified using the 10th Revision of the International Statistical Classification of Diseases (ICD-10). The duration of death follow-up was calculated from the date of the initial interviews until the date of each participant’s death.

### Statistical analyses

Our research followed the NHANES analytic guidelines, considering complex sampling designs and sampling weights. Day 1 dietary sample weights (WTDRD1) were applied in all analyses. Continuous variables are reported as means and standard errors (SEs), while categorical variables are presented as proportions. Live microbe consumption from diet was sorted into two categories: a low dietary live microbe intake group and a medium-high dietary live microbe intake group. Depression status was divided into two groups: a depression group and a non-depression group.

NHANES data are collected through a complex survey design, including stratification, clustering, and weighting. Parameter estimation and statistical inference are primarily based on the survey design rather than the data distribution. In complex survey design, the core of statistical methods lies in adjusting for weights to reflect the characteristics of the target population, rather than relying on the assumption of normal distribution. Therefore, for complex survey data, the normality assumption is not a necessary condition for parametric tests. The weighted Student’s t-test is a commonly used method in complex survey data analysis. It incorporates survey weights (e.g., WTDRD1 or WTMEC2YR) and design variables (e.g., strata SDMVSTRA and clusters SDMVPSU) into the calculations, ensuring that the results are representative. So student’s t-test was employed to compare continuous variables, while the chi-square test was used for categorical variables. Participants were grouped according to depressive symptoms and dietary live microbe intake to estimate mortality risks and explore joint associations using multivariable Cox proportional hazards regression models adjusted for a consistent set of covariates. Three models were developed to facilitate statistical inference. Model 1 was unadjusted. Model 2 included Model 1 along with sex, age, race and BMI. Model 3 expanded upon Model 2 by including uric acid, neutrophil count(Neu), white blood cell count (WBC), HbA1c, blood urea nitrogen, hemoglobin (HB), cardiovascular disease (CVD), diabetes mellitus (DM), and hypertension.

A Cox proportional hazards regression analysis was performed in this cross-sectional study to calculate the adjusted hazard ratios (aHR) and 95% confidence intervals (95% CIs) for the associations between dietary live microbe intake, PHQ-9 scores, and all-cause, cardiovascular disease (CVD), and non-CVD mortality among cancer patients. To investigate the potential mediating effect of the PHQ-9 score between dietary live microbe intake and all-cause cancer mortality, a mediation analysis was conducted.

This analysis included estimating the overall effect of dietary live microbe intake on all-cause mortality (α), the effect of dietary live microbe intake on the PHQ-9 score (β1), and the effect of the PHQ-9 score on all-cause mortality (β2). The direct effect of dietary live microbe intake on all-cause mortality was calculated using the formula 1-β1*β2/ɑ= 81.6%, 95%CI, 70.8-92.5%. The indirect effect of dietary live microbe intake on all-cause mortality was calculated using the formula β1*β2/ɑ= 18.4%, 95%CI, 7.5-29.2%. Statistical analyses were performed using R Studio version 4.2.0. A significance level of *p* < 0.05 was set for the statistical analyses.

## Results

### Characteristics of the participants

A total of 2,899 cancer patients were included in this study. Among the participants, the weighted median age was 62.87 years, with 86.48% (*n* = 2,011) identifying as white and 56.39% (*n* = 1,527) as female. Significant differences were observed between survivors and those who deceased in various factors, including age, race, blood urea nitrogen, albumin, neutrophil count, hemoglobin, uric acid, total bilirubin, hypertension, and diabetes mellitus (all *p* < 0.0001). Additionally, significant differences were noted in sex, HbA1c levels, and dietary live microbe intake (*p* < 0.005). However, no significant differences were found in body mass index (BMI) or PHQ-9 scores. Detailed participant information is summarized in Table 1. Significant differences were observed between survivors and those who deceased in various cancer types (*p* < 0.0001) (Supplementary Table S1).

**Table 1:** Baseline characteristics of study participants. Mean ± SEs for continuous variables: *P* value was calculated by weighted student’s t test. Number (%) for categorical variables: P value was calculated by weighted chi-square test. Abbreviations: BMI, body mass index; Edu, education; Neu, neutrophile granulocyte; CVD, cardiovascular disease; DM, diabetes mellitus; IFG, impaired fasting glucose; IGT, impaired glucose tolerance; PHQ9, 9-item Patient Health Questionnaire

Variable	Total	Death/No	Death/Yes	*P* value
Age(years)	62.865(0.377)	60.674(0.417)	73.020(0.497)	< 0.0001***
Sex(%)				< 0.001**
Female	1527(56.392)	1234(58.369)	293(47.230)	
Male	1372(43.608)	939(41.631)	433(52.770)	
BMI (kg/m2 )	28.993(0.162)	29.134(0.175)	28.343(0.392)	0.063
Race(%)				< 0.0001***
White	2011(86.476)	1437(85.810)	574(89.560)	
Black	384( 4.919)	287(4.607)	97(6.368)	
Other	504( 8.605)	449(9.583)	55(4.072)	
Blood Urea Nitrogen(mg/dl)	15.943(0.175)	15.339(0.194)	18.739(0.391)	< 0.0001***
Albumin(g/L)	42.167(0.081)	42.388(0.091)	41.145(0.177)	< 0.0001***
Neu(%)	59.915(0.247)	59.483(0.291)	61.920(0.499)	< 0.0001***
Hemoglobin(g/dl)	14.035(0.038)	14.113(0.041)	13.675(0.079)	< 0.0001***
Uric acid(mg/dl)	5.483(0.039)	5.404(0.044)	5.847(0.066)	< 0.0001***
HbA1c(%)	5.786(0.018)	5.751(0.020)	5.947(0.043)	< 0.001**
Bilirubin total (mg/dl)	0.650(0.008)	0.634(0.009)	0.725(0.015)	< 0.0001***
Hypertension				< 0.0001***
No	1037(41.669)	861(45.488)	176(23.970)	
Yes	1862(58.331)	1312(54.512)	550(76.030)	
CVD				< 0.0001***
No	2210(81.299)	1764(85.216)	446(63.144)	
Yes	689(18.701)	409(14.784)	280(36.856)	
DM				< 0.0001***
DM	793(22.409)	538(20.085)	255(33.179)	
IFG	175( 6.304)	139(6.685)	36(4.541)	
IGT	144( 4.150)	94(3.753)	50(5.986)	
No	1787(67.137)	1402(69.477)	385(56.294)	
Live microbes intake				0.003*
No	2029(75.499)	1556(76.842)	473(69.276)	
Yes	870(24.501)	617(23.158)	253(30.724)	
PHQ-9				0.603
No	2607(91.049)	1945(90.895)	662(91.765)	
Yes	292( 8.951)	228(9.105)	64(8.235)	

### Associations of dietary live microbe intake with all-cause, CVD and non-CVD mortality of cancer

Table 2 illustrates the association between dietary live microbe intake and all-cause, CVD, and non-CVD mortality in cancer patients, adjusted for various models. Regarding all-cause mortality, Model 1 produced an adjusted hazard ratio (HR) of 0.704 (95% CI, 0.565–0.877; *P* = 0.002) after adjusting for several factors. In a similar vein, Model 2 yielded an HR of 0.715 (95% CI, 0.580–0.882; *P* = 0.002). Furthermore, Model 3 indicated an HR of 0.741 (95% CI, 0.602–0.912; *P* = 0.005). In terms of CVD mortality, Model 1 produced an adjusted HR of 0.744 (95% CI, 0.526–1.052; *P* = 0.094) after accounting for various factors. Model 2 also yielded an HR of 0.783 (95% CI, 0.538–1.150; *P* = 0.216). Additionally, Model 3 indicated an HR of 0.750 (95% CI, 0.510–1.104; *P* = 0.145). For non-CVD mortality, Model 1 yielded an HR of 0.680 (95% CI, 0.530–0.871; *P* = 0.002) after adjusting for various factors. Similarly, Model 2 produced an HR of 0.675 (95% CI, 0.535–0.850; *P* < 0.001). Furthermore, Model 3 indicated an HR of 0.687 (95% CI, 0.545–0.866; *P* = 0.001). For cancer mortality, Model 3 indicated an HR of 0.833 (95% CI, 0.612–1.135; *P* = 0.247) (Supplementary Table S2).We analyzed the data by maintaining the original three-group categorization (low, medium, and high) in Table S5 (Supplementary Table S5). The results showed a consistent trend, with medium and high intake groups both associated with reduced mortality risk compared to the low intake group. However, the high intake group alone did not show a statistically significant difference from the low intake group in terms of all-cause and CVD mortality.

**Table 2:** Cox regression analysis demonstrating associations of dietary live microbes intake and mortality

		Model 1		Model 2		Model 3	
Dietary Live Microbe Intake		95%CI	*P* value	95%CI	*P* value	95%CI	*P* value
	**All-cause**						
	Low	ref.		ref.		ref.	
	Med-Hi	0.704(0.565,0.877)	0.002	0.715(0.580,0.882)	0.002	0.741(0.602,0.912)	0.005
	**CVD**						
	Low	ref.		ref.		ref.	
	Med-Hi	0.744(0.526,1.052)	0.094	0.783(0.538,1.150)	0.216	0.750(0.510,1.104)	0.145
	**Non-CVD**						
	Low	ref.		ref.		ref.	
	Med-Hi	0.680(0.530,0.871)	0.002	0.675(0.535,0.850)	< 0.001	0.687(0.545,0.866)	0.001

Model 1: Live microbe intake only

Model 2: Model 1, Sex, Age, Race, BMI

Model 3: Model 2, Uric Acid, WBC, Neu, HbA1c, HB, Blood Urea Nitrogen, CVD, DM and Hypertension

### Associations of depression with all-cause, CVD and non-CVD mortality of cancer

Table 3 outlines the association between depression and all-cause, CVD, and non-CVD mortality in cancer patients, adjusted for various models. Compared to individuals without depression (reference group), Model 1 showed an adjusted hazard ratio (HR) of 0.853 (95% CI, 0.573–1.271; *P* = 0.435), after accounting for various factors. Likewise, Model 2 produced an HR of 1.899 (95% CI, 1.342–2.688; *P* < 0.001). Additionally, Model 3 indicated an HR of 1.780 (95% CI, 1.281–2.473; *P* < 0.001) for all-cause mortality. For CVD mortality, Model 3 reported an HR of 1.468 (95% CI, 0.752–2.864; *P* = 0.261). In terms of non-CVD mortality, Model 3 revealed an HR of 1.901 (95% CI, 1.249–2.793; *P* = 0.001). For cancer mortality, Model 3 indicated an HR of 1.165 (95% CI, 0.632–2.148; *P* = 0.624) (Supplementary Table S3).

**Table 3:** Cox regression analysis demonstrating associations of depression and mortality

		Model 1		Model 2		Model 3	
Depression							
	**All-cause**	95%CI		95%CI		95%CI	
	No	ref.	*P* value	ref.	*P* value	ref.	*P* value
	Yes	0.853(0.573,1.271)	0.435	1.899(1.342,2.688)	< 0.001	1.780(1.281,2.473)	< 0.001
	**CVD**						
	No	ref.		ref.		ref.	
	Yes	0.611(0.304,1.228)	0.167	1.692(0.881,3.248)	0.114	1.468(0.752,2.864)	0.261
	**Non-CVD**						
	No	ref.		ref.		ref.	
	Yes	0.931(0.597,1.453)	0.754	2.077(1.403,3.074)	< 0.001	1.901(1.249,2.793)	0.001

Model 1: PHQ-9 only

Model 2: Model 1, Sex, Age, Race, BMI

Model 3: Model 2, Uric Acid, WBC, Neu, HbA1c, HB, Blood Urea Nitrogen, CVD, DM and Hypertension

### Joint associations of dietary live microbe intake and depression with all-cause, CVD and non-CVD mortality of cancer

Table 4 illustrates the joint association between dietary live microbe intake and depression with all-cause, CVD, and non-CVD mortality in cancer patients, adjusted for various models. The joint analyses indicated that low dietary live microbe intake was linked to the highest rates of all-cause and non-CVD mortality in cancer patients, independent of depression.We conducted a joint analysis of depression, dietary live microbe intake, and mortality among cancer survivors. Surprisingly, the joint analysis revealed that the combination of depression and low dietary live microbe intake was associated with increased risks of all-cause mortality(HR, 3.122; 95% CI, 1.734–5.619; *P* < 0.001), CVD mortality(HR, 3.547; 95% CI, 1.189–10.585; *P* = 0.023), and non-CVD mortality (HR, 3.236; 95% CI, 1.699–6.117; *P* = 0.004). For cancer mortality, Model 3 indicated an HR of 1.662 (95% CI, 0.767-3.600; *P* = 0.198) (Supplementary Table S4).

**Table 4:** Cox regression analysis demonstrating associations of dietary live microbes intake, depression and mortality

		Model 1		Model 2		Model 3	
		95%CI	*P* value	95%CI	*P* value	95%CI	*P* value
**All-cause**							
	Non-depression + Med-High	ref.		ref.		ref.	
	Non-depression + Low	1.473(1.171,1.853)	< 0.001	1.348(1.084,1.675)	0.007	1.297(1.041, 1.617)	0.021
	Depression + Med-High	0.897(0.578,1.392)	0.627	1.656(1.088,2.522)	0.019	1.532(1.029, 2.281)	0.036
	Depression + Low	1.029(0.511,2.072)	0.937	3.341(1.793,6.228)	< 0.001	3.122(1.734, 5.619)	< 0.001
	*P* for trend		0.339		< 0.0001		< 0.001
**CVD**							
	Non-depression + Med-High	ref.		ref.		ref.	
	Non-depression + Low	1.385(0.956,2.006)	0.085	1.196(0.810, 1.765)	0.369	1.249(0.834, 1.871)	0.281
	Depression + Med-High	0.586(0.227,1.513)	0.270	1.221(0.518, 2.881)	0.648	1.087(0.480, 2.462)	0.842
	Depression + Low	0.802(0.318,2.022)	0.639	4.364(1.538,12.385)	0.006	3.547(1.189,10.585)	0.023
	*P* for trend		0.83		0.071		0.1
**Non-CVD**							
	Non-depression + Med-High	ref.		ref.		ref.	
	Non-depression + Low	1.539(1.189,1.993)	0.001	1.448(1.135,1.848)	0.003	1.391(1.084, 1.786)	0.010
	Depression + Med-High	1.017(0.614,1.685)	0.947	1.939(1.189,3.161)	0.008	1.705(1.048, 2.774)	0.032
	Depression + Low	1.089(0.491,2.413)	0.833	3.316(1.628,6.753)	< 0.001	3.236(1.699,6.117)	< 0.001
	*P* for trend		0.22		< 0.0001		< 0.001

Model 1: Dietary live microbes intake and PHQ-9

Model 2:Model 1, Sex, Age, Race, BMI

Model 3: Model 2, Uric Acid, WBC, Neu, HbA1c, HB, Blood Urea Nitrogen, CVD, DM and Hypertension

### Mediation analysis for the association between dietary live microbe intake and all-cause mortality of cancer

**Fig. 2:**
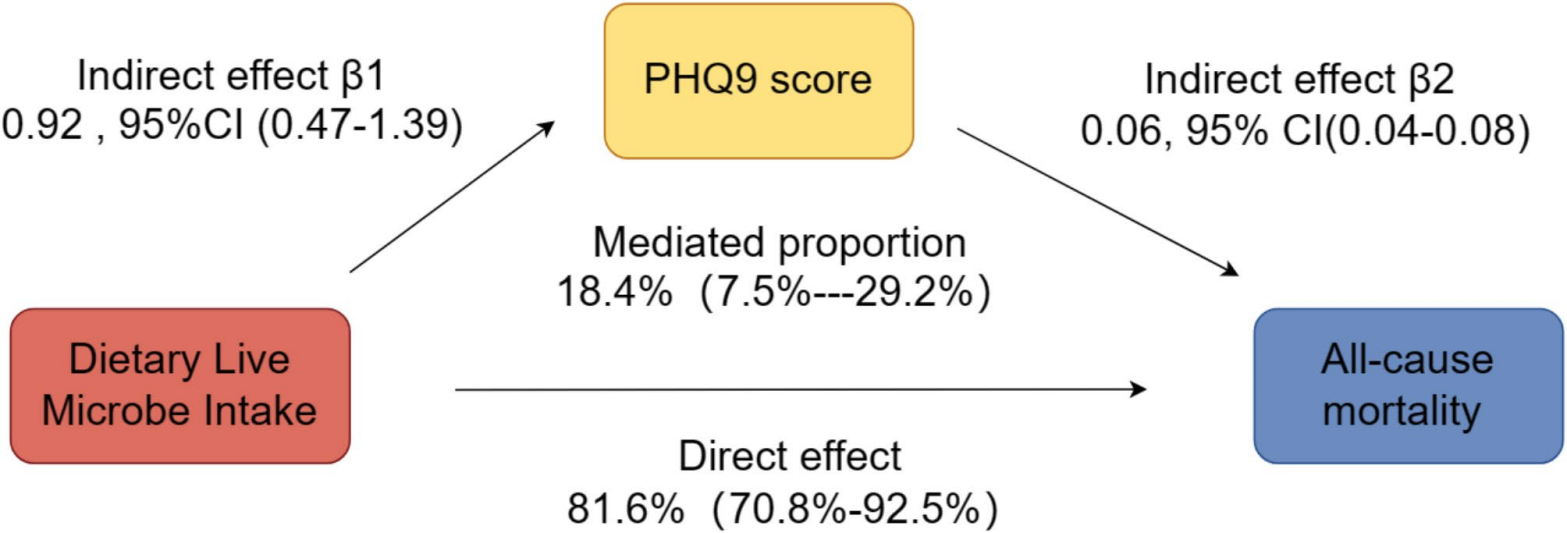
Mediation analysis for the association between dietary live microbe intake and all-cause mortality of cancer

The mediation analysis assessed the role of the PHQ-9 in mediating the relationship between dietary live microbe intake and all-cause mortality in cancer patients.The PHQ-9 demonstrated a mediating effect in this association. The mediation proportions was 18.4% (95% CI, 7.5%--29.2%) (Fig. 2).

## Discussion

To our knowledge, this is the first study to examine both the individual and combined effects of dietary live microbe intake and depression on mortality outcomes using a nationally representative sample of cancer patients. The study found that low dietary live microbe intake and depression were associated with an increased risk of non-CVD and all-cause cancer mortality, respectively. In the joint analysis, the combination of low dietary live microbe intake and depression significantly heightened the risk of non-CVD and all-cause cancer mortality. The PHQ-9 score exhibited a notable mediating effect in the relationship between dietary live microbe intake and all-cause cancer mortality. Humans consume live microbes in various ways, particularly through fermented foods (foods produced via controlled microbial growth and enzymatic transformations of food components) and probiotics (live microorganisms that, when taken in sufficient amounts, provide health benefits to the host) [33, 34]There is a hypothesis that consuming live dietary microbes may offer health benefits [35]. However, conducting randomized, double-blind, placebo-controlled trials to assess the effects of live dietary microbes is challenging due to the limited variety of tested microbes and foods available. While systematic reviews have previously focused on specific health outcomes related to certain dietary microbes [36–39], a comprehensive review of the evidence regarding the health outcomes associated with the broader consumption of dietary microbes is necessary.A knowledge gap remains regarding the relationship between dietary-induced changes in the microbiome and long-term consequences, such as cancer development. Several review articles have highlighted studies investigating cancer in relation to specific dietary patterns [25–27], fermented foods [40–42], probiotics [43]and particular microbial strains [44–46]. However, fewer studies have focused on the relationship between dietary live microbe intake and the mortality risk associated with all types of cancers. Therefore, our study further supports the beneficial role of medium-high live microbe intake in reducing the mortality risk associated with cancer, potentially offering valuable insights for updating live microbe intake recommendations.

Depression frequently co-occurs with cancer in patients. Cancer patients are reported to have a higher risk of developing depression [47]. Previous studies have identified depression as a predictor of increased mortality among cancer patients [20, 21, 48]. Recent studies have demonstrated that depression is significantly associated with an increased risk of both cancer-specific mortality and all-cause mortality in cancer patients [49, 50]. In line with previous research, our study found that depression elevates the risk of all-cause and non-CVD mortality in cancer patients, indicating that depression may affect disease progression.

Several factors may account for our findings, including lifestyle, behavioral, and biological factors. First, cancer patients with depression may adopt unhealthy lifestyles [51]. Second, nonadherence to treatment is a significant issue. For example, cancer patients may struggle to adhere to chemotherapy and other adjunct treatments, potentially resulting in poor prognosis [52]. Additionally, mortality may also be attributed to non-natural causes of death, particularly suicide [53].

To further investigate the relationship between dietary live microbe intake, depression, and cancer mortality, we conducted a mediation analysis that revealed a significant mediating role of the PHQ-9 score. Increased dietary live microbe intake significantly lowered PHQ-9 scores, and lower PHQ-9 scores were associated with reduced all-cause cancer mortality. The mechanisms involving the microbiota-gut-brain axis and inflammation may help explain our findings. Numerous studies have highlighted the significant connections between dietary live microbe intake and depression. A nationwide population-based study found that high dietary live microbe intake can help prevent and alleviate symptoms in individuals with depression [54]. A population-based randomized clinical trial (RCT) showed that probiotics effectively reduced anxiety and depressive symptoms when used alongside antidepressants for eight weeks [55]. Emerging research indicates that certain strains of probiotics, particularly those within the Lactobacillus and Bifidobacterium genera, may influence mood regulation and help alleviate depressive symptoms [56, 57]. Furthermore, animal models receiving gut microbiota transplants from patients with depression displayed anxiety and depressive-like behaviors [58, 59].Several mechanisms may clarify how dietary live microbe affect depression. Evidence suggests that live microorganisms in the diet can reach the gut, colonize this area, interact with resident microbes, influence the type and composition of the gut microbiota, and potentially exert positive effects [11, 60, 61]. Gut microbiota are crucial components of the brain-gut axis [62]. Gut microbiota significantly influence depression through the microbiota-gut-brain axis. Gut microbes regulate the activity of the hypothalamic-pituitary-adrenal axis and neurotransmitter release, which helps alleviate depression-like behaviors in animals [63]. Various mediators, such as short-chain fatty acids, tryptophan catabolites, and microbial-associated molecular patterns, are involved in the interactions between gut microbiota and the brain [64, 65]. Certain biomarkers elevated in inflammatory and chronic disease states are also found to be increased in depression.Interleukin-6 (IL-6), tumor necrosis factor-α (TNF-α), and cortisol have been linked to depression and are associated with symptom severity [66–69]. Other studies have shown that patients receiving anti-inflammatory therapies experience an improvement in depressive symptoms [70, 71]. Significant evidence suggests that cytokines and glucocorticoids are linked to a poorer prognosis in cancer patients [72–79].IL-6 and TNF-α play roles in all stages of tumor development, including formation, progression, and metastasis [77]. Abnormal cortisol levels may hinder DNA repair pathways and promote cancer cell growth [72]. These common inflammatory mechanisms in cancer and depression have led researchers to observe that depressive symptoms frequently precede a cancer diagnosis [80]. This trend has been especially noted in pancreatic and lung cancers, as well as in cancers that cause malignant hypercalcemia [81]. Considering the mechanisms of the microbiota-gut-brain axis and inflammation, it is not surprising that depression is associated with increased all-cause and non-CVD mortality in cancer. Our study notably demonstrated a mediating effect of depression on the relationship between dietary live microbe intake and all-cause cancer mortality.

From a public health standpoint, we recommend a comprehensive strategy for cancer patients that addresses both dietary live microbe intake and mental health. Enhancing dietary live microbe intake and proactively addressing depression could contribute to lower mortality rates. Considering the interconnectedness of nutrition, mental health, and chronic diseases, healthcare policymakers should incorporate these factors into their planning and develop holistic management strategies tailored specifically for cancer patients.

### Strengths and limitations

#### Strengths

Our study has several strengths. Initially, this is the first study to explore the joint effects of dietary live microbe intake and depression with mortality of all types of cancers. Second, we utilized the sample weights, clustering, and stratification to estimate appropriate variance and ensure national representation of the US population. The nationally representative sample of US cancer in this study encompassed multiple races to enhance the generalizability of our findings to other cancer populations. Third, we controlled a series of covariates including the demographic characteristics, comorbidities and blood examination, which would help to reduce the potential confounding bias. Last, we conducted mediation analyses to validate the association between live microbes intake and all-cause mortality of cancer.

#### Limitations

This study has several limitations. First, while the survey was administered by professional interviewers and the questionnaire has been widely used in various studies, the diagnosis of depressive symptoms relied on self-reported data, which may introduce information bias. Second, the 24-hour dietary recall data may suffer from inaccuracies due to recall bias, and the levels of dietary live microbe can be influenced by transportation, storage, and cooking methods. Third, Sanders’ classification system for dietary live microbe may be less accurate than direct measurement.However, direct measurement is time-consuming and costly, which limits its feasibility. Fourth, the broad categorization of microbial diets could lead to estimation errors in assessing actual microbial intake. Fifth, it is hard to differentiate the treatment backgrouds which could potentially influence the outcomes, because Nhanes database does not record treatment of tumor patients. Sixth, since our study is based on the US population, and regional differences in dietary habits and gut microbiome composition do present potential challenges when it comes to generalizing the findings to other regions. Seventh, despite adjustments for confounding factors, unknown variables may still exist. Eighth, Additionally, while this study focuses on the impact of depression on cancer survivor mortality, it does not consider other psychological and social factors, such as anxiety, stress, and social support, which may also significantly influence mortality outcomes among cancer survivors. Ninth, this study did not directly explore specific mechanisms of the microbiota-gut-brain axis, such as the potential roles of short-chain fatty acids, inflammatory markers (e.g., IL-6, TNF-α), or neurotransmitter changes (e.g., serotonin, dopamine) in the observed associations. Future research should validate these hypothesized mechanisms through experimental designs, such as animal models or clinical trials, to provide a more comprehensive understanding of the complex relationships between dietary live microbe intake, depression, and cancer mortality. Therefore, future research should involve a large prospective cohort study consider all of above limitations.

## Conclusion

Our study found that low dietary live microbe intake was linked to increased all-cause and non-CVD mortality in cancer patients, independent of depression. The combination of low dietary live microbe intake and depression was associated with the highest risk of all-cause and non-CVD mortality in cancer patients. The PHQ-9 scores showed a notable mediating effect on the relationship between dietary live microbe intake and all-cause mortality in cancer patients. These findings underscore the importance of adopting medium-high dietary live microbe habits and addressing depression to reduce cancer mortality risk. Future prospective cohort studies are needed to further explore and validate this association.

## Electronic supplementary material

Below is the link to the electronic supplementary material.

Supplementary Material 1

Supplementary Material 2

Supplementary Material 3

Supplementary Material 4

Supplementary Material 5

## Data Availability

The original contributions presented in the study are included in the article, further inquiries can be directed to the corresponding authors.
