# Correction: Joint association of dietary live microbe intake and depression with cancer survivor in US adults: evidence from NHANES

**DOI:** 10.1186/s12885-025-14405-4

**Published:** 2025-06-04

**Authors:** Dekui Jin, Tian Lv, Chengying Zhang, Yi Hu

**Affiliations:** 1 Medical School of Chinese PLA, Chinese PLA General Hospital, Beijing, 100853 China; 2 Department of Neurology, Zhuji Affiliated Hospital of Wenzhou Medical University, Shaoxing, Zhejiang China; 3 Department of General Practice, The Third Medical Center of Chinese PLA General Hospital, Beijing, 100039 China; 4 Department of Oncology, The Fifth Medical Center of PLA General Hospital, Beijing, 100853 China

**Correction: BMC Cancer 25, 487 (2025)**

10.1186/s12885-025-13699-8

Following publication of the original article [1], the authors identified an error in the author names. The given names and family names were erroneously transposed.

The author group has been updated above and the original article [1] has been corrected.

## References

[1] Jin D, Lv T, Zhang C, et al. Joint association of dietary live microbe intake and depression with cancer survivor in US adults: evidence from NHANES. BMC Cancer. 2025;25:487. 10.1186/s12885-025-13699-8. 40098072 10.1186/s12885-025-13699-8 PMC11912725

