# Supplementary material for: Joint association of dietary live microbe intake and depression with cancer survivor in US adults: evidence from NHANES
Source: BMC Cancer. 2025 Mar 17;25:487. doi: 10.1186/s12885-025-13699-8 (PMC11912725; doi:10.1186/s12885-025-13699-8)
Supplement: Supplementary file 1 — Supplementary Material 1 [file 12885_2025_13699_MOESM1_ESM.doc]

| Variable | Total | Death/No | Death/Yes | P value |
| --- | --- | --- | --- | --- |
| Cancer Type |  |  |  | < 0.0001*** |
| Blood Cancer | 94( 3.035) | 70(2.880) | 24(3.753) |  |
| Bone Cancer | 9( 0.309) | 4(0.203) | 5(0.800) |  |
| Brain Cancer | 13( 0.378) | 10(0.331) | 3(0.593) |  |
| Breast Cancer | 428(14.448) | 321(14.033) | 107(16.369) |  |
| Lung Cancer | 66( 1.997) | 39(1.538) | 27(4.123) |  |
| Melanoma | 185( 8.144) | 141(8.483) | 44(6.574) |  |
| Urogenital Cancer | 567(12.633) | 391(11.345) | 176(18.599) |  |
| Skin Cancer | 690(30.901) | 524(31.721) | 166(27.099) |  |
| Soft Tissue Cancer | 3( 0.172) | 2(0.123) | 1(0.401) |  |
| Reproductive Cancer | 390(13.733) | 349(15.462) | 41( 5.725) |  |
| Thyroid Cancer | 60( 2.013) | 55(2.372) | 5(0.349) |  |
| Gastrointestinal Cancer | 238( 6.633) | 147( 5.564) | 91(11.585) |  |
| Other Cancer | 156( 5.604) | 120(5.943) | 36(4.032) |  |

Table S1. Baseline characteristics of study participants’ cancer type.

Mean±SEs for continuous variables: P value was calculated by weighted Student’s t test. Number (%) for Categorical variables: P value was calculated by weighted chi-square test.
