# Supplementary material for: Joint association of dietary live microbe intake and depression with cancer survivor in US adults: evidence from NHANES
Source: BMC Cancer. 2025 Mar 17;25:487. doi: 10.1186/s12885-025-13699-8 (PMC11912725; doi:10.1186/s12885-025-13699-8)
Supplement: Supplementary file 2 — Supplementary Material 2 [file 12885_2025_13699_MOESM2_ESM.doc]

|  |  | Model 1 | | Model 2 | | Model 3 | |
| --- | --- | --- | --- | --- | --- | --- | --- |
| Dietary Live Microbe Intake |  | 95%CI | *P* value | 95%CI | *P* value | 95%CI | *P* value |
|  | **Cancer** |  |  |  |  |  |  |
|  | Low | ref. |  | ref. |  | ref. |  |
|  | Med-Hi | 0.794(0.595,1.061) | 0.119 | 0.800(0.607,1.055) | 0.114 | 0.833(0.612,1.135) | 0.247 |

Table S3-1 : Cox regression analysis demonstrating associations of dietary live microbes intake and cancer related mortality.

Model 1: Live microbe intake only.

Model 2: Model 1, Sex, Age, BMI,Race.

Model 3: Model 2, Uric Acid, WBC, Neu,HbA1c,HB,Blood Urea Nitrogen, CVD, DM and Hypertension.
