# Supplementary material for: Joint association of dietary live microbe intake and depression with cancer survivor in US adults: evidence from NHANES
Source: BMC Cancer. 2025 Mar 17;25:487. doi: 10.1186/s12885-025-13699-8 (PMC11912725; doi:10.1186/s12885-025-13699-8)
Supplement: Supplementary file 3 — Supplementary Material 3 [file 12885_2025_13699_MOESM3_ESM.doc]

|  |  | Model 1 | | Model 2 | | Model 3 | |
| --- | --- | --- | --- | --- | --- | --- | --- |
| Depression |  |  | |  | |  | |
|  | **Cancer** |  |  |  |  |  |  |
|  | No | ref. | *P* value | ref. | *P* value | ref. | *P* value |
|  | Yes | 0.702(0.393,1.256) | 0.234 | 1.361(0.751,2.465) | 0.309 | 1.165(0.632,2.148) | 0.624 |

Table S3-2: Cox regression analysis demonstrating associations of depression and cancer related mortality.

Model 1: PHQ-9 only

Model 2: Model 1, Sex, Age, BMI,Race

Model 3: Model 2, Uric Acid, WBC, Neu, HbA1c, HB, Blood Urea Nitrogen, CVD, DM and Hypertension.
