# Supplementary material for: Joint association of dietary live microbe intake and depression with cancer survivor in US adults: evidence from NHANES
Source: BMC Cancer. 2025 Mar 17;25:487. doi: 10.1186/s12885-025-13699-8 (PMC11912725; doi:10.1186/s12885-025-13699-8)
Supplement: Supplementary file 4 — Supplementary Material 4 [file 12885_2025_13699_MOESM4_ESM.doc]

|  |  | Model 1 | | Model 2 | | Model 3 | |
| --- | --- | --- | --- | --- | --- | --- | --- |
| Dietary Live Microbe Intake |  | 95%CI | *P* value | 95%CI | *P* value | 95%CI | *P* value |
|  | **All-cause** |  |  |  |  |  |  |
|  | Low | ref. |  | ref. |  | ref. |  |
|  | Med | 0.745(0.591,0.939) | 0.013 | 0.687(0.555,0.851) | <0.001 | 0.726(0.588, 0.895) | 0.003 |
|  | High | 0.645(0.488,0.853) | 0.002 | 0.769(0.586,1.008) | 0.057 | 0.768(0.581, 1.017) | 0.065 |
|  |  |  | 0.02 |  | 0.062 |  | 0.069 |
|  |  |  |  |  |  |  |  |
|  | **CVD** |  | |  | |  | |
|  | Low | ref. |  | ref. |  | ref. |  |
|  | Med | 0.814(0.566,1.170) | 0.266 | 0.754(0.509,1.117) | 0.159 | 0.758(0.512,1.122) | 0.166 |
|  | High | 0.648(0.398,1.054) | 0.081 | 0.853(0.521,1.398) | 0.528 | 0.736(0.438,1.238) | 0.248 |
|  |  |  | 0.077 |  | 0.533 |  | 0.256 |
|  | **Non-CVD** |  | | | | | |
|  | Low | ref. |  | ref. |  | ref. |  |
|  | Med | 0.716(0.552,0.927) | 0.011 | 0.647(0.513,0.815) | <0.001 | 0.668(0.529, 0.844) | <0.001 |
|  | High | 0.630(0.457,0.868) | 0.005 | 0.727(0.531,0.996) | 0.047 | 0.721(0.520, 1.000) | 0.050 |
|  |  |  | 0.005 |  | 0.054 |  | 0.055 |

Table S2: Cox regression analysis demonstrating associations of dietary live microbes intake in three-group and mortality.

Model 1: Live microbe intake only.

Model 2: Model 1, Sex, Age, BMI,Race.

Model 3: Model 2, Uric Acid, WBC, Neu,HbA1c,HB,Blood Urea Nitrogen, CVD, DM and Hypertension.
