# Supplementary material for: Joint association of dietary live microbe intake and depression with cancer survivor in US adults: evidence from NHANES
Source: BMC Cancer. 2025 Mar 17;25:487. doi: 10.1186/s12885-025-13699-8 (PMC11912725; doi:10.1186/s12885-025-13699-8)
Supplement: Supplementary file 5 — Supplementary Material 5 [file 12885_2025_13699_MOESM5_ESM.doc]

|  |  | Model 1 | | Model 2 | | Model 3 | |
| --- | --- | --- | --- | --- | --- | --- | --- |
|  |  | 95%CI | *P* value | 95%CI | *P* value | 95%CI | *P* value |
| **Cancer** |  |  |  |  |  |  |  |
|  | Non-depression+Med-High | ref. |  | ref. |  | ref. |  |
|  | Non-depression+Low | 1.322(0.961,1.817) | 0.086 | 1.270(0.935,1.726) | 0.126 | 1.174(0.841,1.637) | 0.346 |
|  | Depression+Med-High | 0.757(0.361,1.590) | 0.463 | 1.297(0.602,2.794) | 0.507 | 1.057(0.480,2.330) | 0.891 |
|  | Depression+Low | 0.741(0.336,1.636) | 0.458 | 1.843(0.853,3.981) | 0.120 | 1.662(0.767,3.600) | 0.198 |
|  | *P* for trend |  | 0.866 |  | 0.088 |  | 0.296 |

Table S3-3: Cox regression analysis demonstrating associations of dietary live microbes intake, depression and cancer related mortality.

Model 1: Dietary live microbes intake and PHQ-9.

Model 2:Model 1, Sex, Age, BMI, Race.

Model 3: Model 2, Uric Acid, WBC, Neu,HbA1c,HB,Blood Urea Nitrogen, CVD, DM and Hypertension.
